# Supplementary material for: Population Variation and Phylogeography of Cherry Blossom (Prunus conradinae) in China
Source: Plants (Basel). 2024 Mar 28;13(7):974. doi: 10.3390/plants13070974 (PMC11013036; doi:10.3390/plants13070974)
Supplement: Supplementary file 1 [file plants-13-00974-s001.zip › plants-2902936-supplementary.pdf]

Supplementary Information for

# Population Variation and Phylogeography of Cherry Blossom (*Prunus conradinae*) in China

**This file includes:**

**Table S1. Score coefficients of the initial three principal components in relation to 19 environmental variables.**

| Climatic factors | Principal component | | |
|---|---|---|---|
| | PC1 | PC2 | PC3 |
| bio4 | 0.02 | 0.83 | 0.40 |
| bio6 | 0.73 | -0.33 | 0.55 |
| bio7 | -0.33 | 0.82 | 0.29 |
| bio9 | 0.80 | -0.36 | 0.44 |
| bio11 | 0.70 | -0.49 | 0.50 |
| bio12 | 0.83 | 0.46 | -0.26 |
| bio16 | 0.85 | 0.03 | -0.46 |
| bio17 | 0.67 | 0.69 | -0.01 |
| bio18 | 0.63 | -0.33 | -0.60 |
| bio19 | 0.70 | 0.66 | -0.05 |

**Table S2. Adaptive area of *P. conradinae* under different climatic conditions （×104 km2）**

| Period | Climatic scenario | Low permissive area | Middle permissive area | High permissive area | Extremely permissive area | Total |
|---|---|---|---|---|---|---|
| current | | 74.79 | 65.32 | 28.43 | 1.46 | 170.00 |
| 2050 | RCP2.6 | 83.29 | 53.57 | 28.49 | 2.85 | 168.20 |
| | RCP4.5 | 71.53 | 60.26 | 38.97 | 4.68 | 175.44 |
| | RCP6.0 | 76.79 | 50.55 | 36.90 | 4.45 | 168.69 |
| | RCP8.5 | 74.15 | 55.71 | 35.18 | 3.33 | 168.37 |
| 2070 | RCP2.6 | 78.19 | 66.16 | 32.50 | 2.41 | 179.26 |
| | RCP4.5 | 77.78 | 57.44 | 29.76 | 2.02 | 167.00 |
| | RCP6.0 | 84.30 | 54.86 | 34.59 | 6.28 | 180.03 |
| | RCP8.5 | 68.82 | 63.03 | 40.89 | 4.70 | 177.44 |

**Table S3 Haplotypes sequence variation of three cpDNA fragments was detected in *P. conradinae*.**

| Haplotype | Nucleotide position | | | | | | | | | | | | | | | | | | | | | | (No.) |
|---|---|---|---|---|---|---|---|---|---|---|---|---|---|---|---|---|---|---|---|---|---|---|---|
| | MatK | | | | | | | | | TrnL-F | | | | | | | | TrnD-E | | | | | |
| | 5 | 7 | 0 | 0 | 0 | 0 | 0 | 0 | 2 | 3 | 3 | 3 | 5 | 5 | 0 | 0 | 0 | 0 | 2 | 4 | 5 | 5 | |
| | 7 | 3 | 0 | 2 | 3 | 3 | 8 | 9 | 8 | 1 | 4 | 6 | 1 | 2 | 2 | 3 | 4 | 4 | 1 | 1 | 0 | 9 | |
| | 9 | 8 | 6 | 4 | 3 | 4 | 2 | 0 | 6 | 2 | 9 | 6 | 6 | 2 | 6 | 2 | 0 | 6 | 7 | 5 | 8 | 0 | |
| Hap1 | A | A | C | A | G | T | G | A | G | T | C | T | G | T | C | C | G | G | C | A | A | T | 74 |
| Hap2 | · | · | T | · | · | · | C | · | · | · | · | · | · | · | · | · | · | · | · | · | · | · | 4 |
| Hap3 | · | · | · | · | · | · | · | T | · | · | · | · | · | · | · | · | · | · | · | · | · | · | 17 |
| Hap4 | · | · | · | · | · | · | · | · | · | · | · | A | · | · | · | · | · | · | · | · | · | · | 9 |
| Hap5 | · | · | · | G | · | · | · | · | · | · | · | A | · | · | · | · | · | · | · | · | · | · | 3 |
| Hap6 | · | · | · | · | · | · | · | · | · | · | · | A | · | · | · | T | C | · | · | · | · | · | 8 |
| Hap7 | · | · | · | · | · | · | · | · | · | G | · | · | · | · | · | · | · | · | · | · | · | · | 60 |
| Hap8 | · | · | · | · | · | · | · | · | · | G | · | · | · | · | · | · | C | · | · | · | · | · | 6 |
| Hap9 | · | · | · | · | · | · | · | · | · | G | · | · | · | · | · | · | · | · | · | · | · | C | 6 |
| Hap10 | · | · | · | · | T | C | · | · | · | · | · | · | · | · | · | · | · | · | · | · | · | · | 2 |
| Hap11 | · | · | · | · | · | · | · | · | · | · | G | · | · | G | · | · | C | · | · | · | G | · | 2 |
| Hap12 | · | · | · | · | · | · | · | · | · | · | G | · | · | · | · | · | C | · | · | · | G | · | 4 |
| Hap13 | · | · | · | · | · | · | · | · | · | · | G | · | · | · | · | T | · | A | · | · | G | · | 5 |
| Hap14 | · | · | · | · | · | · | · | · | A | · | G | · | A | · | · | · | · | · | · | · | G | · | 5 |
| Hap15 | · | · | · | · | · | · | · | · | · | · | G | · | · | · | · | · | · | A | · | · | G | · | 23 |
| Hap16 | · | C | · | · | · | · | · | · | · | G | G | · | · | · | · | · | · | · | T | · | G | · | 7 |
| Hap17 | G | · | · | · | · | · | · | · | · | · | G | · | · | · | T | · | · | · | · | · | G | · | 7 |
| Hap18 | · | · | · | · | · | · | · | · | · | · | G | · | G | G | · | · | · | A | · | G | G | · | 2 |

Note: "·" is the same as the base of Hap 1 site

**Table S4 NrDNA sequence polymorphisms detected of identifying 11 ribotypes in *P. conradinae*.**

| Ribotype | Nucleotide position | | | | | | | | | | | | | | | | | | | （No.） |
|---|---|---|---|---|---|---|---|---|---|---|---|---|---|---|---|---|---|---|---|---|
| | ITS | | | | | | | | | | | | | | | | | | | |
| | 0<br>7<br>3 | 1<br>2<br>4 | 1<br>5<br>7 | 1<br>6<br>7 | 3<br>6<br>8 | 3<br>7<br>4 | 3<br>7<br>5 | 3<br>9<br>1 | 3<br>9<br>6 | 4<br>0<br>1 | 4<br>3<br>6 | 4<br>6<br>9 | 4<br>8<br>5 | 4<br>8<br>7 | 5<br>3<br>9 | 5<br>7<br>1 | 5<br>7<br>2 | 5<br>7<br>3 | 5<br>7<br>5 | |
| R1 | T | T | G | T | C | G | C | G | C | A | A | T | C | A | C | T | A | G | C | 47 |
| R2 | A | · | · | · | · | · | · | · | · | · | · | · | · | · | G· | · | · | · | · | 16 |
| R3 | · | · | · | · | · | · | · | · | · | · | G | · | · | · | · | · | · | · | · | 28 |
| R4 | · | · | · | · | T | A | A | T | T | · | G | G | · | C | · | · | · | · | · | 85 |
| R5 | · | · | · | · | · | A | A | T | T | · | G | G | · | C | · | · | · | · | · | 22 |
| R6 | · | · | · | G | T | A | A | T | T | · | G | G | · | C | · | C | T | A | G· | 4 |
| R7 | · | · | · | G | T | A | A | T | · | · | G | G | · | C | · | · | · | · | · | 2 |
| R8 | · | · | · | G | · | A | A | T | T | · | G | G | · | C | · | · | · | · | · | 3 |
| R9 | · | · | · | G | T | A | A | T | T | · | G | G | · | C | · | · | · | · | · | 4 |
| R10 | · | · | · | · | T | A | A | T | · | · | G | G | · | C | · | · | · | · | · | 2 |
| R11 | · | C | T | · | · | · | · | · | · | G | G | G | T | C | · | · | · | · | · | 25 |

Note: "·" is the same as the base of R1 site

**Table S5.** Summary of cpDNA and nrDNA-based divergence time estimation by Bayesian.

| Node | Median value (Ma) | 95% HPD |
|---|---|---|
| N1: # Rosaceae（Subfamily Amygdaloideae + Subfamily Rosoideae） | 92.17 | 92.07-92.29 |
| N2: # (Tribe Maleae + Tribe Spiraeeae) + Tribe Amygdaleae | 75.61 | 75.39-75.76 |
| Tribe Maleae + Tribe Spiraeeae | 67.26 | 67.07-67.44 |
| Tribe Maleae | 44.96 | 44.77-45.12 |
| N3: #*Prinsepia* + ((*Laurocerasus*+(*Padus* + *Maddenia*))+ (*Amygdalus* + (*Prunus* + *Armeniaca*))+*Cerasus*) | 68.53 | 68.27-68.72 |
| N4: # (*Laurocerasus*+(*Padus* + *Maddenia*))+ (*Amygdalus* + (*Armeniaca* + *Prunus*))+*Cerasus* | 49.81 | 49.47-50.0 |
| *Laurocerasus*+(*Padus* + *Maddenia*) | 35.11 | 31.54-38.83 |
| N5: # (*Amygdalus* + (*Armeniaca* + *Prunus*))+Sub.g *Cerasus* | 28.13 | 28.0-28.26 |
| 1. *Prunus mahaleb*+*Cerasus* | 15.25 | 13.89-16.37 |
| 2.(*P. clarofolia* + (*P. pseudocerasus* + *P. scopulorum*)) + *P. conradinae* | 11.71 | 8.09-15.31 |
| 3. *P. conradinae* | 4.38 | 2.38-6.51 |
| 4. *P. conradinae* (Central China + East China) | 3.32 | 1.12-5.17 |
| 5. *P. conradinae* (East China +（Central China+Southwest China) ) | 2.17 | 0.47-4.54 |
| 6. *P. conradinae* (Central China + Southwest China) | 1.1 | 0.11-2.85 |

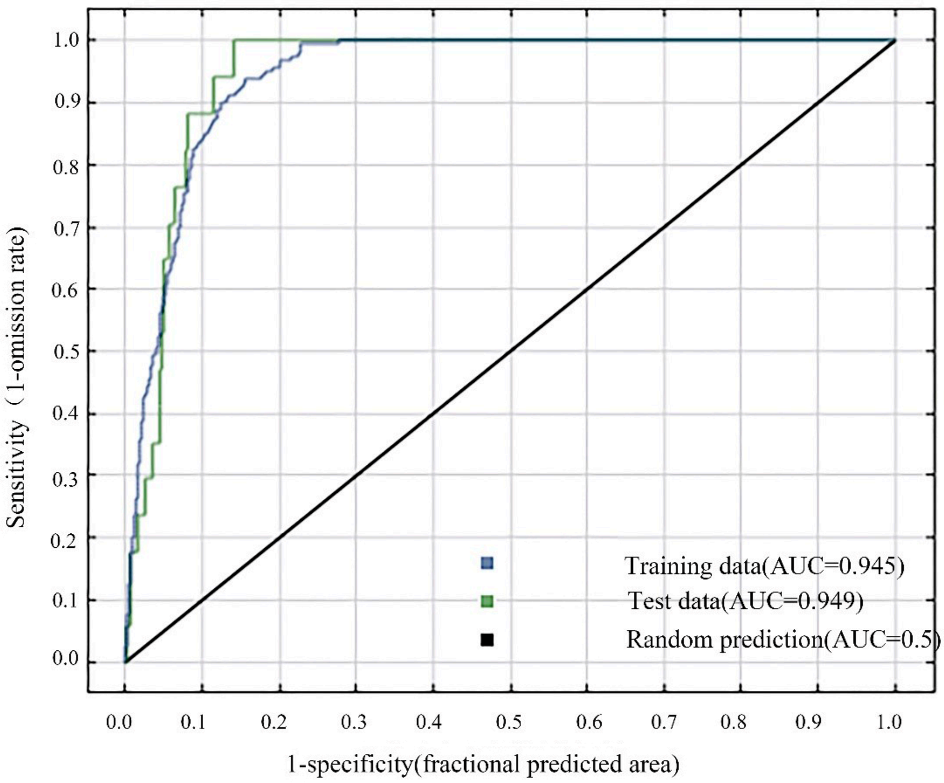


**Fig.S1 ROC curve prediction results in MaxEnt model**

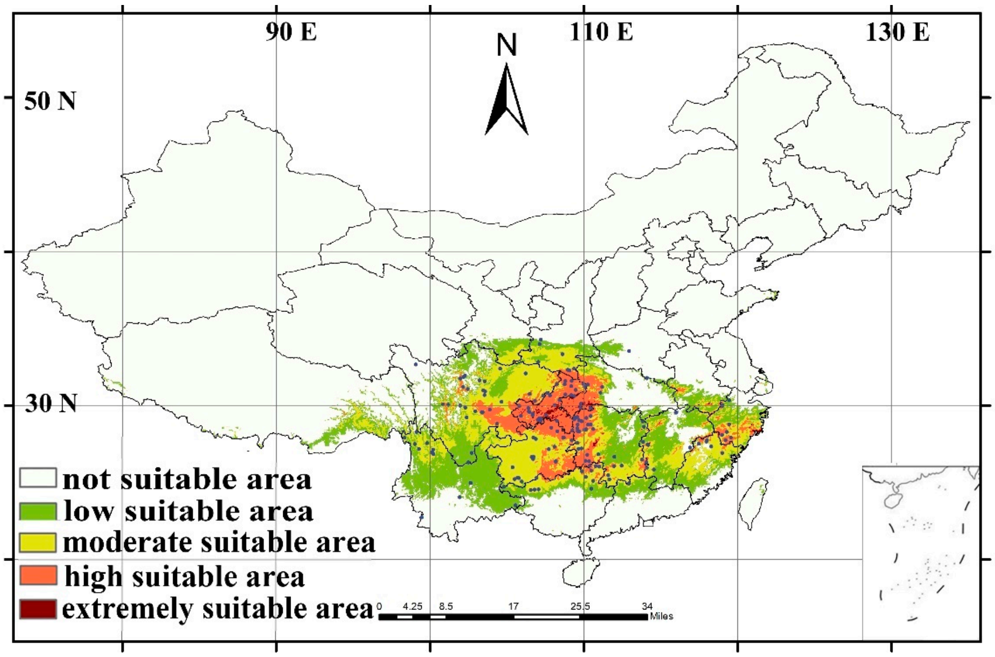


**Fig.S2 Current climate conditions in China offer potential habitat for *P. conradinae.***

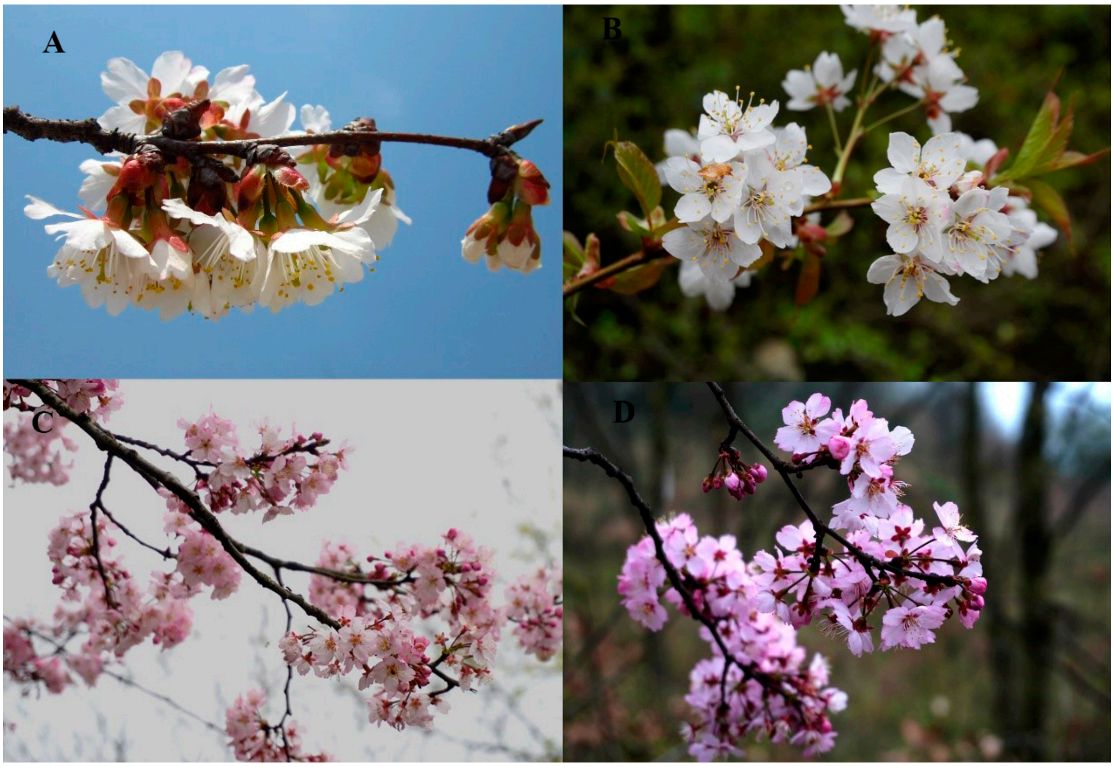


**Fig.S3** ***P. conradinae*** **variation in color.**

**( A-B.** ***P. conradinae;*****C-D.** ***P. conradinae*** **var.** ***ruburm*****)**

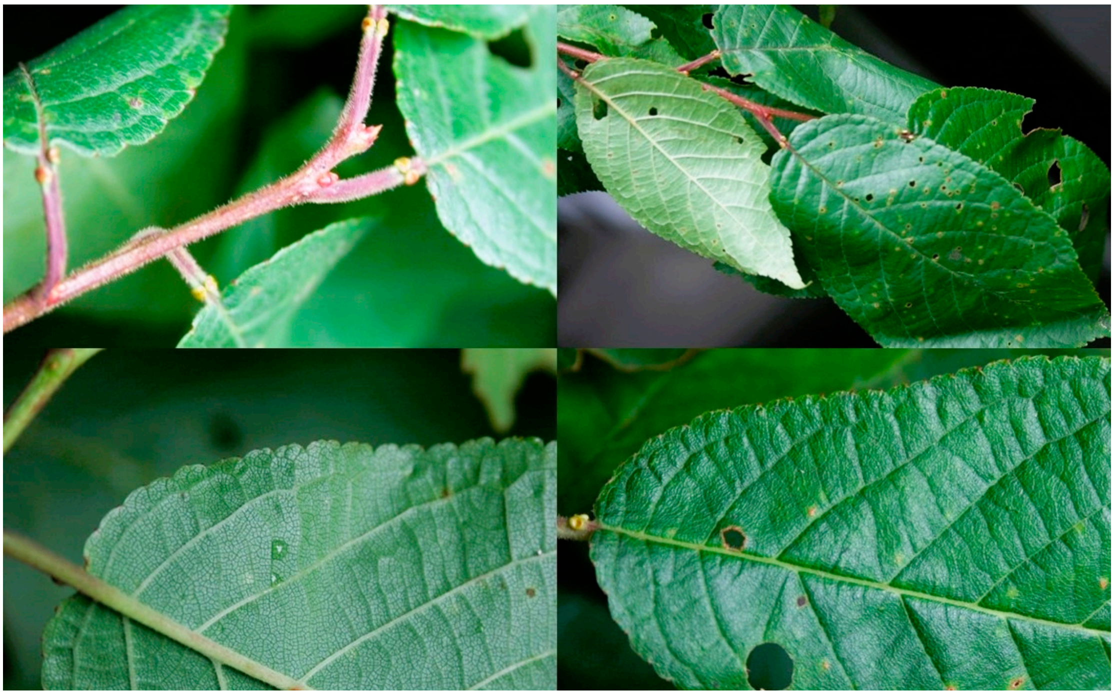

**Fig.S4 Variation of coat of leaf in** ***P. conradinae*****. (*****P. conradinae*** **var.** ***pubescens*****)**

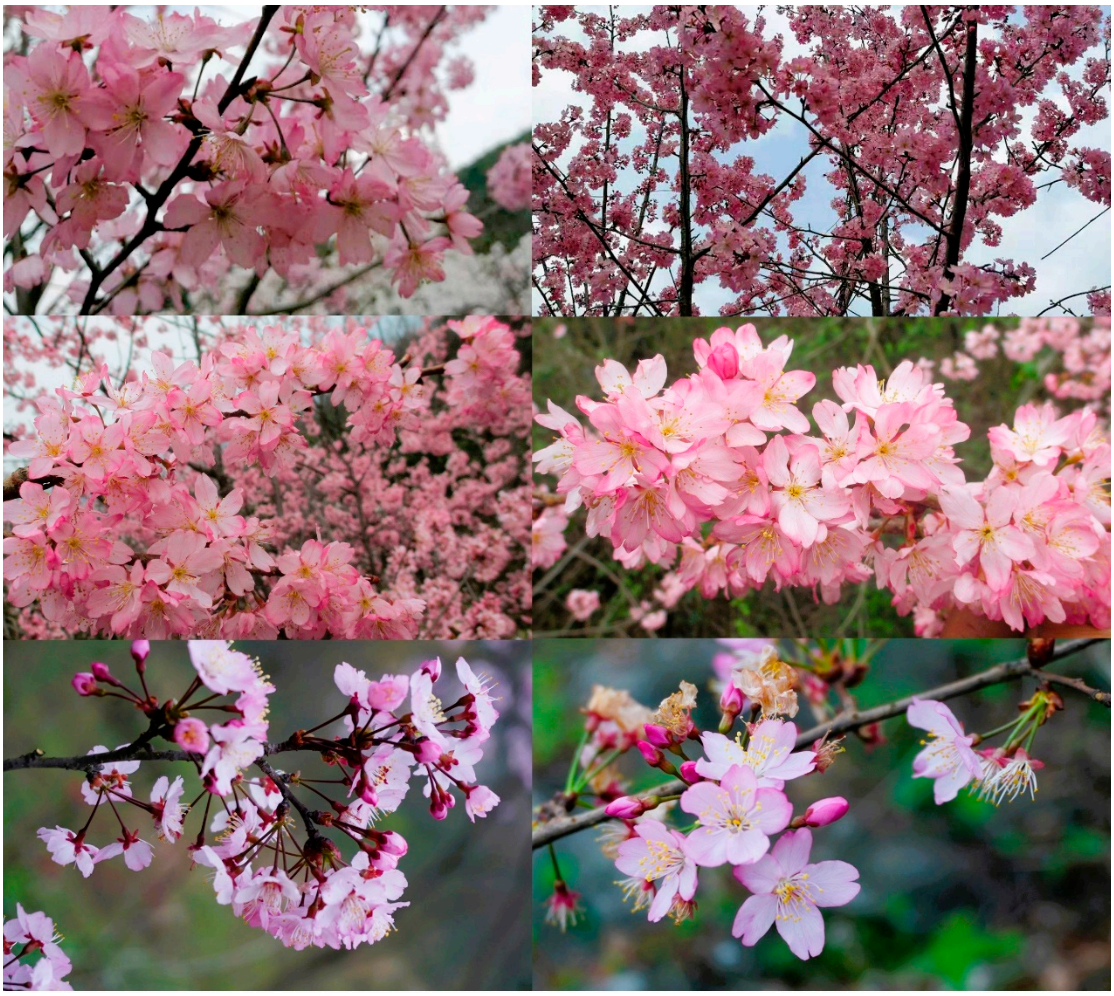

**Fig.S5** ***P. conradinae*** **var.** ***pubescens*** **in different populations**
